# Differential contributions of human oligosaccharyltransferase complexes OST-A and OST-B to HIV-1 envelope glycoprotein glycosylation

**DOI:** 10.1128/jvi.01481-25

**Published:** 2026-03-04

**Authors:** Tugba Atabey, Ronald Derking, Maddy L. Newby, Joey H. Bouhuijs, Jonne L. Snitselaar, Monique Vink, Yoann Aldon, Joel D. Allen, Max Crispin, Rogier W. Sanders

**Affiliations:** 1 Amsterdam UMC, University of Amsterdam 1234, Amsterdam, the Netherlands; 2 Biological Sciences, University of Southampton 7423, Southampton, United Kingdom; 3 Weill Medical College of Cornell University 5922, New York, New York, USA; Icahn School of Medicine at Mount Sinai, New York, New York, USA

**Keywords:** N-linked glycosylation, oligosaccharyltransferase, STT3B, STT3A, envelope glycoprotein, HIV

## Abstract

N-linked glycosylation of glycoproteins during synthesis in the endoplasmic reticulum (ER) is mediated by oligosaccharyltransferase (OST) complexes OST-A and OST-B that have different catalytic subunits STT3A and STT3B, respectively. OST-A acts cotranslationally, while OST-B adds glycans posttranslationally. While there is redundancy between these two enzymes, it is unclear how they both contribute to glycosylation of the densely glycosylated HIV-1 envelope glycoprotein complex (Env). We found that knocking out STT3A had a profound negative impact on HIV-1 virus production and infectivity, while STT3B ablation had no such effect, suggesting that STT3A is more important than STT3B for Env glycosylation and preserved function. STT3A/3B knockout (KO) affected the neutralization sensitivity to broadly neutralizing antibodies (bNAbs) in a strain-specific manner, with STT3A-KO increasing susceptibility to VRC01 bNAb for the tested HIV-1 strains. In contrast, for the BG505 clade A virus, it conferred increased resistance to glycan-dependent bNAbs 2G12 and PGT128. For other HIV-1 strains, STT3B-KO also led to resistance to glycan-dependent bNAb PGT151. Site-specific glycan analysis of recombinant Env proteins revealed that STT3A-KO reduced glycan occupancy of potential N-linked glycosylation sites (PNGS) more globally than STT3B-KO, with certain acceptor sites, including N234 and N386, showing STT3A dependence. In contrast, STT3B-KO appeared to have a more pronounced effect on gp41 glycosylation, suggesting that PNGS located near the C-terminus are more dependent on STT3B. Defining the roles of the OST-A and OST-B complexes in HIV-1 Env glycosylation may bring critical information for the development of methods to control PNGS glycan occupancy of recombinant glycoprotein immunogens.

**IMPORTANCE:**

HIV-1 envelope glycoprotein (Env) complex is the sole target of broadly neutralizing antibodies (bNAbs), making it the primary focus of vaccine design efforts. The Env glycoprotein is one of the most heavily glycosylated proteins found in nature. However, the contributions of oligosaccharyltransferase (OST) isoforms STT3A and STT3B to Env glycosylation have not been fully characterized. Under-occupancy of potential N-linked glycosylation sites (PNGS) on recombinant Env glycoproteins can elicit off-target immune responses and pose challenges for HIV-1 vaccine development. Understanding and controlling the mechanisms behind PNGS occupancy is therefore critical for rational immunogen design. This study demonstrated that the viral Env glycosylation is mostly controlled by OST-A. Additionally, site-specific glycan analysis of recombinant Env proteins identified several STT3A-dependent sites and confirmed a dominant role of STT3B in C-terminal glycosylation. Our fundamental study contributes novel insights into host-cell-mediated glycosylation and informs the development of methods to regulate PNGS occupancy of Env-based immunogens.

## INTRODUCTION

N-linked glycosylation of polypeptides is crucial for proper folding, stability, protein trafficking, and secretion (1–3). HIV-1 envelope glycoprotein (Env) is among the most heavily glycosylated proteins found in nature, with glycosylation playing a critical role in viral infectivity, immune evasion, and host interactions (4–8). N-linked glycans are attached to potential N-linked glycosylation sites (PNGS) on each protomer, totaling up to 100 PNGS per Env trimer and accounting for approximately half the molecular mass of the external domains of Env. HIV-1 Env gp120 subunits are densely glycosylated with up to 35 PNGS per gp120 protomer, while the gp41 subunits typically harbor 4 PNGS per protomer (7, 8). N-linked glycosylation defines the highly dynamic viral glycan shield and shapes antigenic evolution (7, 9).

PNGS consist of asparagine (N) residues in NxT/Sx sequons, where x is any amino acid except proline. The presence of proline at the x position disrupts this sequon and prevents glycosylation, while residues at the second (x), third (T/S), as well as those flanking the sequon, influence the efficiency of N-linked glycan addition (10, 11). Oligosaccharyltransferase (OST) complexes add glycan precursor molecules (GlcNac_2_-Man_9_-Glc_3_; where GlcNAc, Man, and Glc are N-acetylglucosamine, mannose, and glucose, respectively) from a dolichol-pyrophosphate carrier to the NxT/Sx motifs on newly synthesized proteins in the lumen of the endoplasmic reticulum (ER) (12, 13). OST complexes are heterooligomeric transmembrane enzyme complexes embedded in the ER membrane. In human cells, two different OST complexes are expressed and located in this compartment: OST-A and OST-B. OST-A and OST-B complexes regulate cotranslational and posttranslational glycosylation, respectively (14). OST-A and OST-B contain different catalytic subunits referred to as STT3A and STT3B, respectively, and share a set of non-catalytic subunits—including ribophorin I (Rb1), ribophorin II (Rb2), OST48, DAD1, and OST4—plus complex-specific subunits that are part of the OST complex (i.e., DC2 for OST-A and MagT1 for OST-B) (14–16). STT3A and STT3B enzymes exhibit preferences for certain PNGS motifs. As a consequence, OST isoforms preferentially recognize certain NxT/Sx motifs based on the amino acid context adjacent to PNGS targeted motifs. Additionally, steric hindrance or conformational shielding of N-glycosylation sites can limit the ability of OST isoforms to access and glycosylate certain PNGS (17, 18).

The trimeric HIV-1 Env glycoprotein, which is the sole target for broadly neutralizing antibodies (bNAbs) that arise during the natural infection (19, 20), has become the major focus for HIV-1 vaccine development research (21–23). Env, the sole surface protein on HIV-1 virions, is the only virion-associated protein modified by glycosylation; therefore, disrupting glycan addition is unlikely to affect any other viral components (24, 25). Functional Env trimers are derived from gp160 polypeptide precursor chains, which are cleaved into gp120 and gp41 subunits by host proteases, associated into type I fusion glycoprotein trimers, and mediate viral entry into host cells (26, 27). The N-linked glycans that decorate the Env trimer play crucial roles in the viral life cycle, such as binding to lectin receptors, Env protein folding, trafficking between cellular compartments, and immune escape by shielding underlying conserved protein epitopes (7, 20, 28, 29). The glycan addition and composition of recombinant HIV-1 Env trimers can be different from their viral counterparts. PNGS occupancy at some specific PNGS is generally lower on recombinant Env trimers (8, 30, 31). The absence of glycans at these sites can result in holes in the glycan shield, “glycan holes,” forming neo-epitopes that may play a significant role in shaping the antibody response against Env. Glycan holes can form due to the absence of conserved PNGS. For example, in the BG505 virus and its corresponding recombinant Env trimers, the conserved glycosylation sequons at positions N241 and N289 are not present, which creates an immunodominant glycan hole (32, 33). Moreover, glycan holes can appear through incomplete glycosylation of existing functional PNGS motifs when OST-A and OST-B fail to glycosylate a given PNGS (14, 34, 35). It has been shown that PNGS underoccupancy in hypervariable V1 and V2 loops of soluble BG505 SOSIP.664 trimers, found at the trimer apex, can create artificial glycan holes in some Env protomers, leading to uneven underoccupancy of specific sites across the trimer (8). Similarly, when PNGS at position N611 is underoccupied on BG505 SOSIP soluble immunogens, the resulting antibody responses elicited are able to neutralize viruses lacking a glycan at this position but do not display neutralization activity against wild-type (WT) viruses. Furthermore, nsEMPEM analysis of the sera from BG505 SOSIP vaccinated rhesus macaques revealed the immunodominance of two epitope clusters, the V1/V2/V3 region and the trimer base, of which in particular the latter is underglycosylated (32, 33, 36, 37). These glycan holes may distract from more desirable responses and impede the development of neutralization breadth (32, 33, 38). Understanding the limiting factors behind glycan holes on HIV-1 Env recombinant trimers may provide valuable insights for improving immunogen design and vaccine development.

Recently, the CRISPR/Cas9 gene-editing system was used to generate HEK293-derived knockout (KO) cell lines that are deficient for a single catalytic subunit, STT3A or STT3B (39). In the present study, these KO cell lines were used to study the contribution of STT3A or STT3B to PNGS occupancy on viral and recombinant Env glycoproteins. We studied the impact of STT3A/STT3B-KO on the viral infectivity of diverse HIV-1 strains. The recombinant Env glycoproteins were produced in these cell lines for site-specific glycan analysis to interrogate possible STT3A or STT3B-dependent PNGS. Our results therefore contribute to increasing fundamental understanding of viral glycoprotein dependency on OST and highlight potential paths to explore in order to develop methods to better control glycan occupancy on Env-based and other type 1 fusion immunogens.

## RESULTS

### Knocking out STT3A greatly reduces HIV-1 infectivity

**Fig 1:**
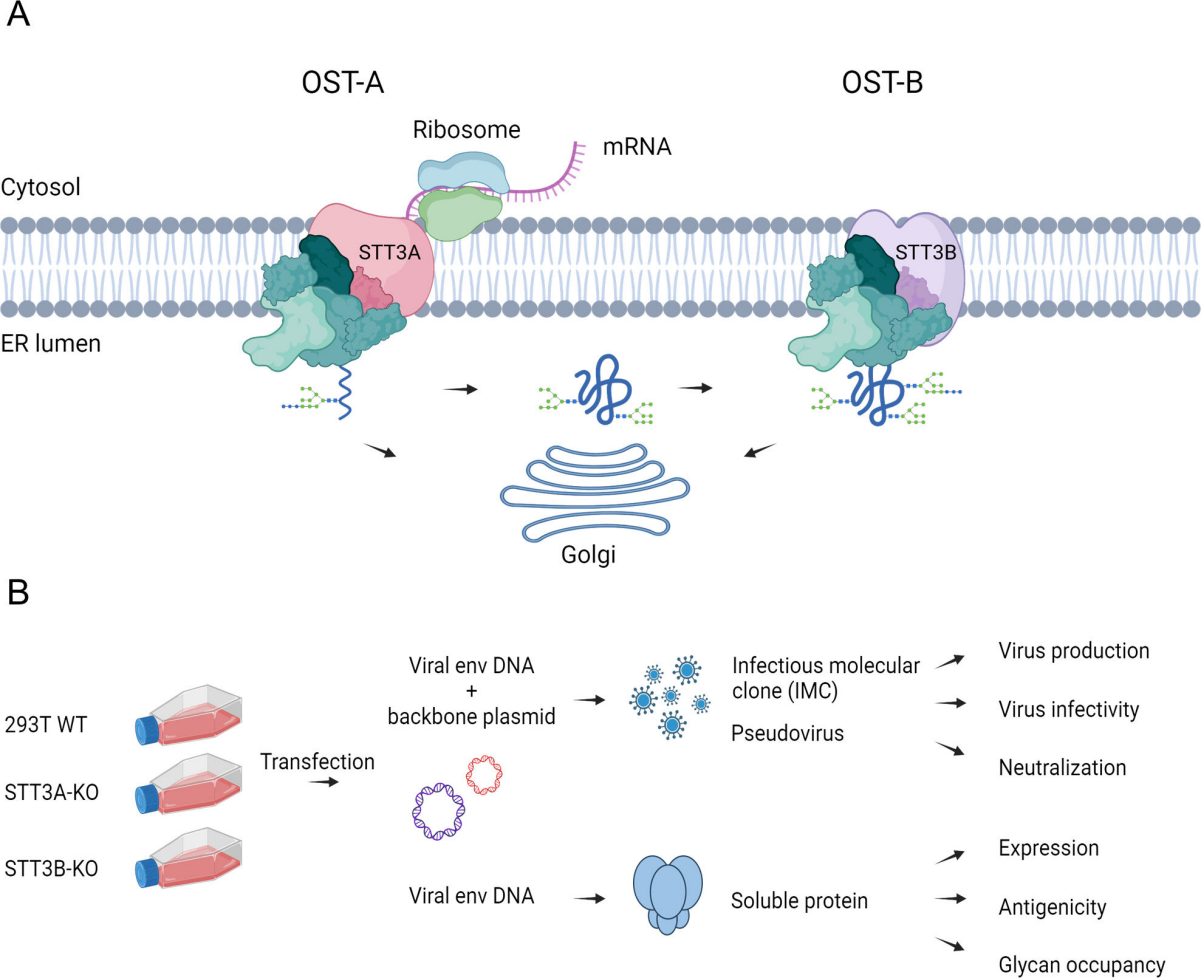
Contribution of OST-A and OST-B complexes to HIV-1 Env glycosylation. (**A**) Schematic representation of OST-A/OST-B-mediated glycosylation in the endoplasmic reticulum (ER). OST-A predominantly catalyzes N-linked glycosylation cotranslationally, adding glycans as the polypeptide is synthesized. In contrast, OST-B acts post-translationally to glycosylate sites that have been skipped by OST-A, ensuring more complete glycan occupancy. Following initial glycosylation in the ER, polypeptides are trafficked through the Golgi apparatus, where further glycan maturation and processing occur. (**B**) Schematic representation of the experimental setup where WT, STT3A-KO, and STT3B-KO HEK293T cells were used to produce HIV-1 virus and recombinant soluble Env trimers. Figures were created using the commercial scientific illustration service BioRender.

**Fig 2:**
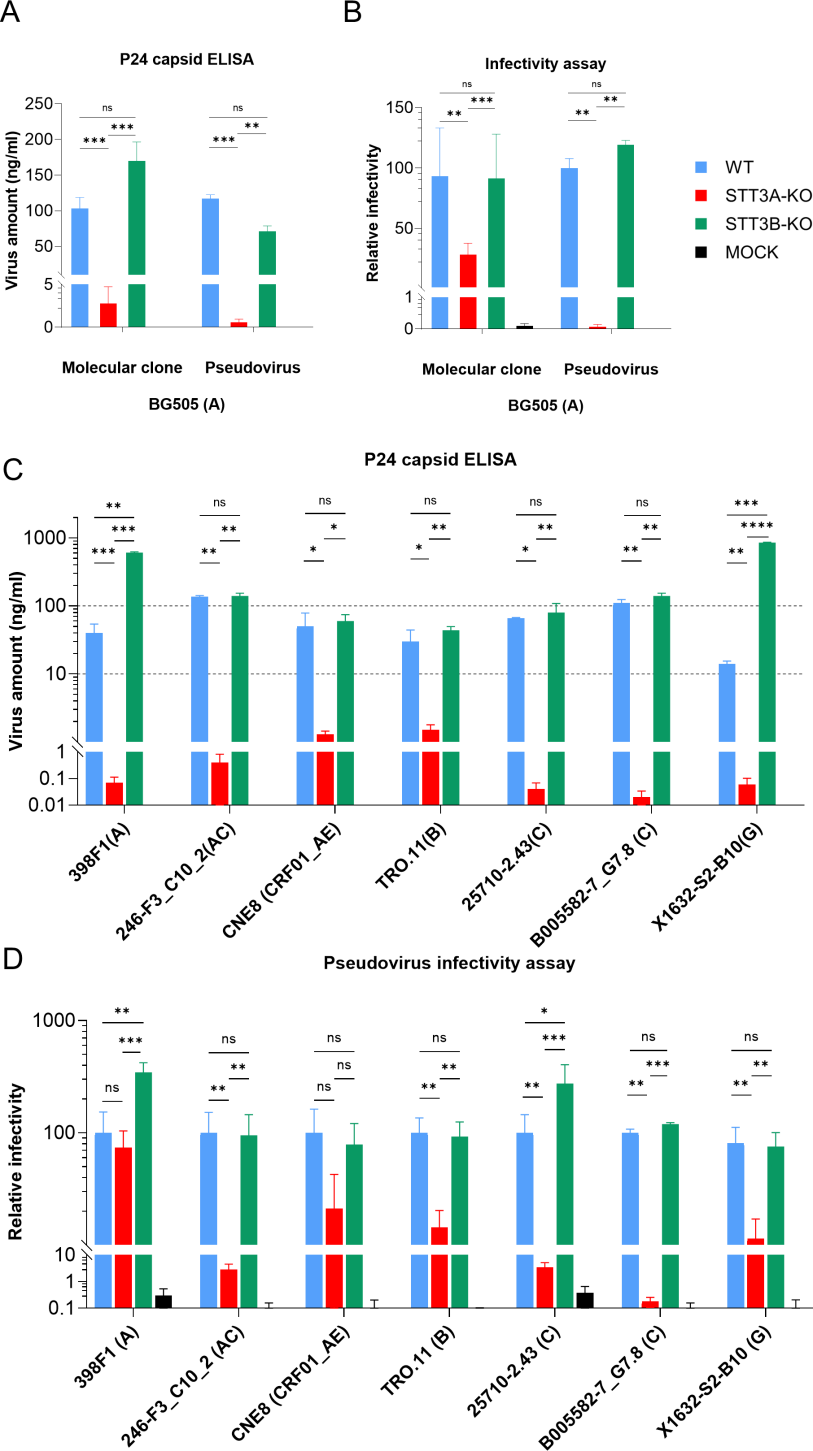
Effect of STT3A and STT3B knockouts on *in vitro* HIV-1 viral production and infectivity. (**A**) Quantification of p24 capsid of BG505 virus and pseudovirus in three different cell lines (blue: wild type [WT], red: STT3A-KO, green: STT3B-KO). The viral productions were quantified by measuring p24 capsid concentrations using a p24 standardized ELISA. (**B**) Relative infectivity of BG505 virus produced in STT3A-KO and STT3B-KO cell lines. Infectivity was assessed using TZM-bl reporter cells, luciferase activity was measured, and infectivity values were normalized to viruses produced in WT cells. (**C**) Quantification of p24 levels by ELISA for pseudotyped HIV-1 viruses derived from different clades/strains and produced in various cell lines. (**D**) Relative infectivity of pseudotyped HIV-1 viruses. The measured luciferase activity was normalized to that of the WT virus. For HIV-1 strains, the clade they belong to is indicated in parentheses. Bars represent the mean ± standard deviation from at least three independent experiments. Statistical significance between conditions was assessed using an unpaired *t*-test. Significance levels are indicated as follows: *, *P* < 0.05; **, *P* < 0.01; ***, *P* < 0.001; ****, *P* < 0.0001.

To explore the roles of the two OST complexes in HIV-1 biology, intact virus from the well-studied BG505 strain was produced in WT, STT3A- and STT3B-KO HEK293T cells as previously described (21) (Fig. 1A and B). A p24 antigen capture ELISA revealed that virus production was severely impaired in STT3A-KO cells (Fig. 2A) (40, 41). This is consistent with previous observations showing that defects in HIV-1 Env biosynthesis and disruptions in N-linked glycosylation that impair Env incorporation can interfere with virus production and egress from infected or producer cells (42–44). We then assessed the virus infectivity of the BG505 strain by measuring its TCID_50_ using the TZM-bl reporter assay, where this modified cell line can be infected by HIV-1 thanks to transgenic expression of CD4, CCR5, and CXCR4 receptors and co-receptors necessary for Env-mediated viral entry. Impairment of the OST-A activity by knocking out STT3A caused a dramatic reduction of HIV-1 infectivity *in vitro*. However, the absence of OST-B activity did not have an appreciable impact, suggesting that N-linked glycosylation of HIV-1 Env native protein is more dependent on OST-A than on OST-B (Fig. 2B).

Besides BG505, other HIV-1 strains derived from clades A, B, C, G, and recombinant forms AC and AE were also produced in these cell lines and assessed for virus production and infectivity. While the above studies on the BG505 strain were performed with both intact and pseudovirus, the following studies were performed with only pseudotyped viruses (see Materials and Methods for details). P24 ELISA results, using pseudotyped viruses, demonstrated that STT3A-KO significantly impaired viral production for all strains tested. In contrast, the ablation of STT3B showed no deleterious effect on production and infectivity for the pseudoviruses tested (Fig. 2C and D). Unexpectedly, we observed that for 398F1 (clade A) and X1632-S2-B10 (clade G) strains produced in STT3B-KO pseudoviruses, production was significantly increased (Fig. 2C). When studying the effect of STT3A or STT3B-KO on infectivity of these diverse HIV-1 strains, it appeared that knocking out STT3A significantly reduced infectivity for most strains, with the exception of the 398F1 (clade A) and CNE8 (clade CRF01_AE) pseudoviruses, although we noted a trend toward reduction in infectivity for CNE8 (Fig. 2D). Impairing STT3B had no negative impact on infectivity of any strain. In fact, the infectivity of the 398F1 and 25710-2-43 strains was enhanced when produced in STT3B-KO cells. The increased viral infectivity observed for the 398F1 virus is likely to be a consequence of the enhanced production observed for this pseudovirus. However, the enhanced production of the X1632-S2-B10 strain did not result in enhanced infectivity, while 25710-2-43 infectivity was enhanced when produced in STT3B-KO cells, while virus production was not affected. To more strictly assess relative infectivity per virus particle, infectivity data were generated by using 1 ng p24 as input (Fig. S1A). The data confirmed that while OST-A is critical for infectivity of most virus isolates, OST-B has a more subtle and strain-specific effect on viral production and infectivity.

Next, to study Env incorporation into virions, we performed gp120 and p24 ELISAs on the supernatants of pseudovirus-producing cells. We performed these assays for BG505, 246-F3_C10_2, and TRO.11 viruses (Fig. S1B). These experiments led to the following observations. First, Env content was substantially lower for all three viruses when they were produced in STT3A-KO cells. This was mirrored by a reduced p24 content, although the fold reduction in p24 content was generally smaller than the fold reduction in gp120 content. For both BG505 and 246-F3_C10_2, gp120 content was also reduced when the virus was produced in STT3B-KO cells, although the reduction was not as great as when using STT3A-KO cells. For TRO.11, the gp120 content for STT3B-KO-produced virus was similar to that of the WT virus. The p24 content was only reduced somewhat (less than two-fold) for BG505 produced in STT3B-KO cells. We also normalized Env levels to particle output using the gp120/p24 ratio. These analyses visualized that while p24 levels were reduced in STT3A-KO cells relative to WT, gp120 levels decreased much more strongly. This suggests that a combination of defects in Env incorporation and/or Env stability on released particles, as well as reduced particle production, underlies the reduced infectivity of virus produced in STT3A-KO cells (Fig. S1B).

Finally, we studied Env expression at the cell surface of transfected cells. WT and STT3A/B-KO HEK293T cells were transiently transfected with full-length BG505 Env plasmid and analyzed by surface staining. The Env signal on STT3A-KO and STT3B-KO cells was comparable to that of WT cells, indicating that Env biosynthesis and transport to the plasma membrane are largely preserved in the absence of STT3A or STT3B (Fig. S1C). These data suggest that the primary defect in STT3A-KO cells is not loss of Env expression, but rather a downstream impairment in virion assembly, Env incorporation, or particle egress.

### Knocking out STT3A affects HIV-1 sensitivity to bNAbs

**Fig 3:**
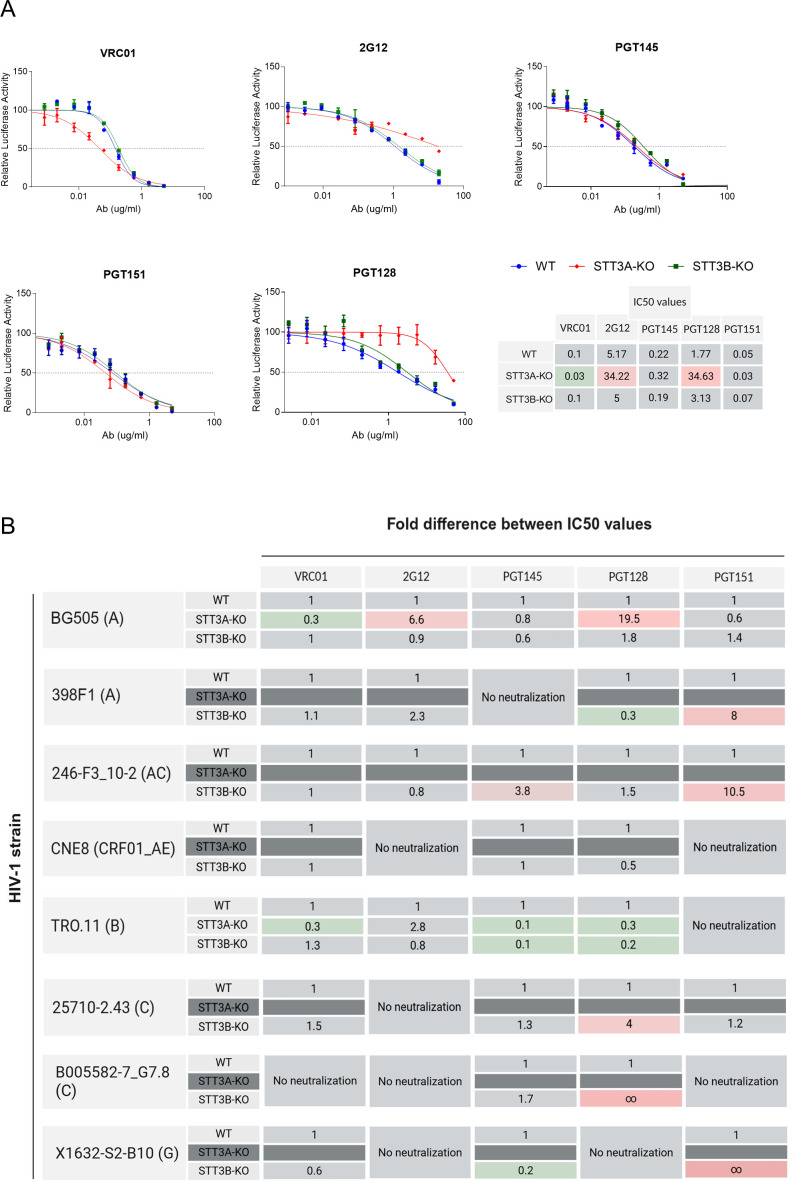
Effect of STT3A and STT3B knockouts on neutralization sensitivity against a panel of bNAbs. (**A**) Neutralization curves and IC₅₀ values for the BG505 virus produced from an intact molecular clone and measured in TZM-bl reporter assay. IC₅₀ values were calculated by fitting neutralization curves to a non-linear regression model and determining the antibody concentration required for 50% inhibition of infection. Changes in neutralization sensitivity are indicated by color coding: green represents a lower IC₅₀ (increased sensitivity to neutralization), pink represents a higher IC₅₀ (increased resistance to neutralization). Bars represent the mean ± standard deviation from at least three independent experiments. (**B**) The effect of STT3A/3B-KO on IC₅₀ values for pseudoviruses from different clades is here reported as a fold change in IC₅₀ compared to pseudoviruses produced in wild-type (WT) cells. The color coding used for the table in panel (**A**) was also applied to panel (**B**). Dark gray indicates that the virus produced in the respective cell lines was not sufficiently infectious to perform neutralization assays. For HIV-1 strains, the clade they belong to is indicated in parentheses.

Next, we assessed whether manipulating OST-A and OST-B activity influenced sensitivity to bNAbs, in particular ones that are heavily dependent on specific Env PNGS glycosylation status. The following bNAbs were selected: PGT145 binding a quaternary epitope at the trimer apex and requiring glycans at N156 and N160; PGT151, targeting a quaternary epitope at the gp120/gp41 interface involving glycans at N611 and N637; PGT128 against the V3-N332 epitope cluster dependent on N295, N301, and N332; 2G12 targeting an epitope that consists exclusively of glycan and involves glycans at N295, N332, N339, and N386; and finally VRC01 directed against the CD4-binding site (CD4bs) and not requiring glycans for binding (45–49). In our assays, we observed that the neutralization sensitivity of the BG505 virus to some bNAbs was substantially altered upon manipulation of OST-A (Fig. 3A). Most notably, STT3A-KO cell line production rendered the BG505 virus more susceptible to VRC01 neutralization. This result could be explained by the STT3A defect resulting in the absence of one or more glycans surrounding the CD4bs, allowing better access to the VRC01 epitope. Indeed, the site-specific occupancy data described below support that hypothesis. Another notable finding consisted of the BG505 virus produced in STT3A-KO cells being more resistant to neutralization by the glycan-dependent bNAbs 2G12 and PGT128, suggesting that one or more of the glycans involved in those targeted epitopes was/were not attached to the protein. The site-specific occupancy data (see below) indicated that the N339 and N386 PNGS were underoccupied as a consequence of STT3A impairment. With regard to quaternary- and glycan-dependent PGT151 and PGT145 bNAbs, no detectable difference in neutralization sensitivity was observed for BG505 virus. We noted that the ablation of STT3B activity had no noticeable effect on BG505 neutralization sensitivity, further emphasizing the critical role of STT3A compared to STT3B in linking glycans to Env glycoprotein in the viral membrane context.

Subsequently, the neutralization sensitivity of our HIV-1 strain panel produced as pseudoviruses in the same cell lines was evaluated. Some strains produced in WT cells were resistant to specific bNAbs, diminishing the value of the comparison between producer cells (Fig. 3B). Only the TRO11 strain (clade B) pseudovirus was sufficiently infective when produced in STT3A-KO cells to proceed with neutralization experiments. TRO11 pseudovirus was more sensitive to VRC01 compared to its WT counterpart, similar to what was observed for BG505 virus. In contrast to the BG505 virus, TRO11 produced in STT3A-KO cells was more sensitive to PGT128 neutralization, suggesting that glycans at positions N295, N301, and N332 that are part of the epitope it targets were attached. We speculate that neighboring glycans may be missing, providing a potential explanation for the enhanced neutralization observed.

STT3B-KO had no effect on the sensitivity of viruses to VRC01 or 2G12. When studying the STT3B-KO impact on PGT128 neutralization, while in some instances increased neutralization sensitivity was observed (i.e., 398F1, TRO.11), it also rendered the 25710-2.43 and B005582-7_G7.8 strains more PGT128 neutralization resistant. These alterations in sensitivity may be attributed to differential glycan occupancy at various sites within and surrounding the epitope. Furthermore, we observed that for several isolates, the PGT151 targeted epitope was affected by STT3B-KO production, making 398F1 (clade A), 246-F3_C10_2 (clade AC), and X1632-S2-B10 (clade G) strains more resistant. We attribute this resistance pattern to the potential absence of interface glycans N611 and/or N637 that are required for efficient PGT151 binding. The effects of STT3A/3B-KO on PGT145 neutralization were also variable: while STT3B-KO increased the sensitivity of TRO-11 and X1632-S2-B10, it rendered the 246-F3_C10_2 strain more resistant. These observations reveal that STT3A and STT3B ablation have highly variable effects on the neutralization sensitivity of different HIV-1 strains to bNAbs. Considering the function of the targeted enzymes knocked out and the bNAbs’ glycan specificities, the observed effects probably depend on whether glycans within the epitopes or around the epitopes targeted are absent. The former would be expected to lead to resistance because critical epitope components are absent, while the latter could lead to enhanced access and therefore enhanced sensitivity.

### Knocking out STT3A or STT3B impacts trimerization

The occupancy of PNGS on recombinant HIV-1 Env trimers has been extensively studied, as has the composition of each glycan (24, 50–56). We produced stabilized (SOSIP.v4.1 [57]) Env trimers of the BG505 strain used above, in WT, STT3A-KO, and STT3B-KO HEK293T cell lines to study occupancy on individual PNGS found in BG505 Env. Two different purification methods were used. First, we used PGT145-antibody affinity chromatography, which is selective for well-folded native-like trimers (58). However, since PGT145 is dependent on glycans, we surmised that its use could bias the glycoforms selected after purification. The second method we used was therefore independent of glycosylation and involved Ni-NTA affinity chromatography, followed by size exclusion chromatography (SEC).

**Fig 4:**
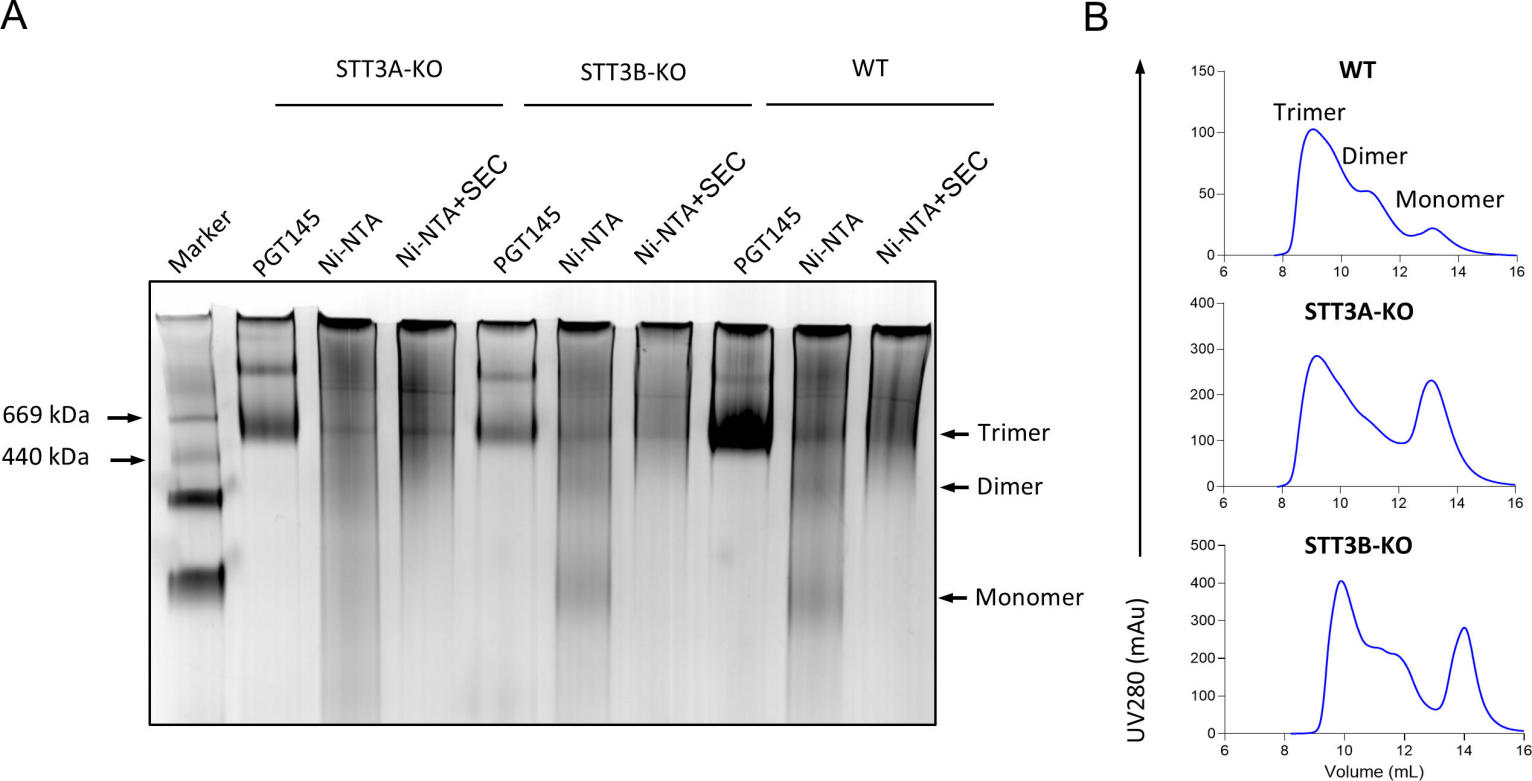
*In vitro* characterization of recombinant Env trimers produced in WT, STT3A-KO, and STT3B-KO HEK293T cell lines. (**A**) Blue native-polyacrylamide gel electrophoresis analysis of Env proteins produced and isolated from different cell lines, stained by Coomassie blue. The PGT145- and Ni-NTA-purified (before and after SEC) Env proteins are shown. The molecular weight of two of the marker bands is indicated on the left end side of the gel picture (thyroglobulin and ferritin), and the expected positions for trimer, dimer, and monomer populations are indicated on the right end side of the picture. (**B**) SEC profiles of Ni-NTA purified Env proteins expressed in three different cell lines. A Superdex 200 10/300 GL column was used. The trimer, dimer, and monomer peaks are indicated. SEC, size exclusion chromatography.

Initial analysis of Ni-NTA-purified material using native gels demonstrated the presence of a heterogeneous mixture comprising trimers, dimers, and monomers, whereas PGT145 immuno-affinity purified material consisted exclusively of trimers (Fig. 4A). This was expected, as tag-based purification methods capture all conformational variants of the protein. When subjected to SEC, a notable increase in the monomeric population was observed for proteins produced in STT3A-KO and STT3B-KO cell lines. This observation suggested that the absence of glycans at some sites may compromise the appropriate trimerization of Env protein. After SEC, the trimer peak was collected, and protein concentrations were measured for all three samples. The trimer yield from STT3A-KO cells was notably lower compared to both STT3B-KO and WT cells (Fig. 4B).

### Knocking out STT3A and STT3B affects binding of specific bNAbs

**Fig 5:**
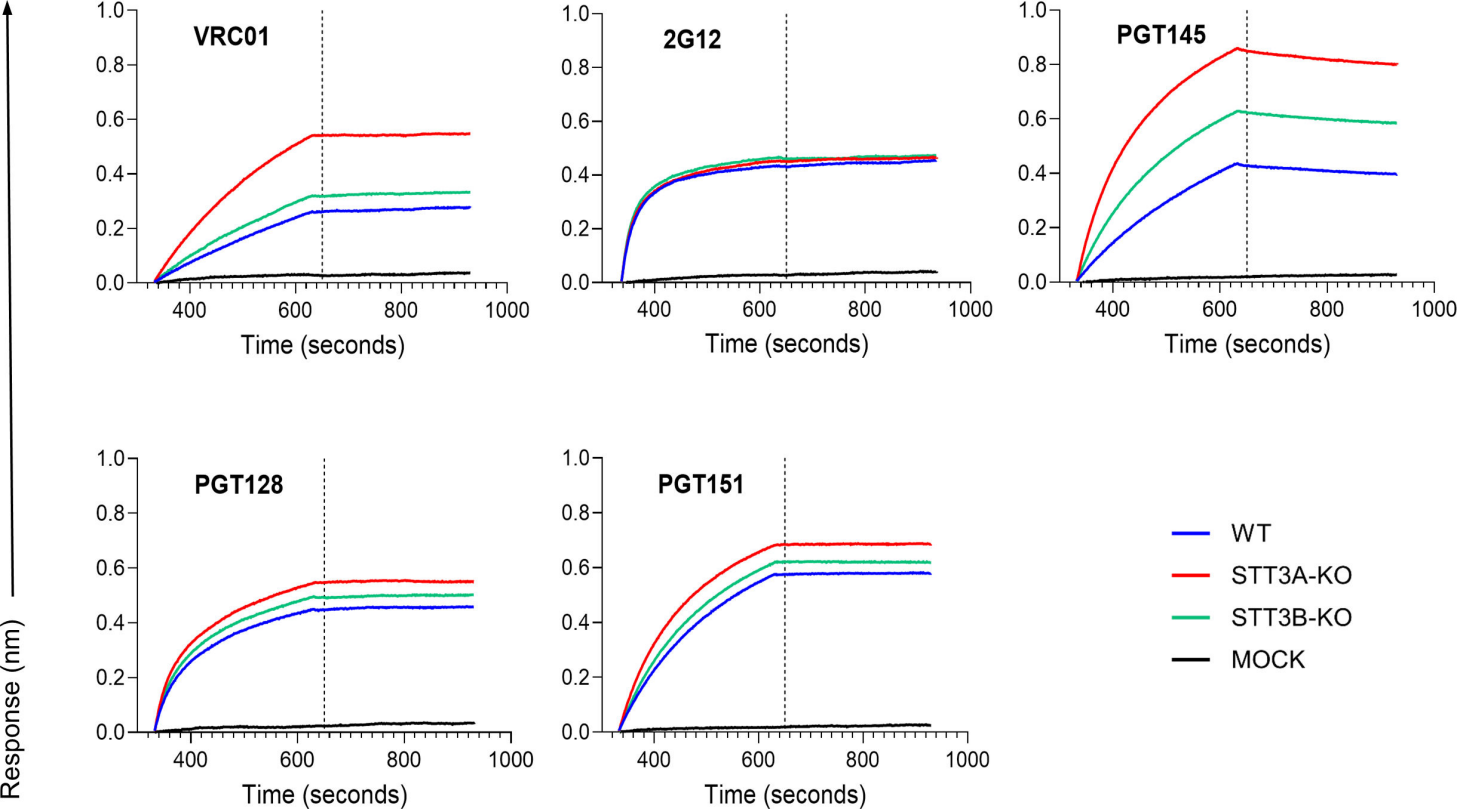
Binding of a panel of mAbs to Env proteins produced in different cell lines. BG505 SOSIP.v4.1 proteins were produced in wild-type (WT), STT3A-KO, or STT3B-KO HEK293T cell lines and purified through immune-affinity chromatography using PGT145. Biolayer interferometry (BLI) measurements were performed on the PGT145-purified trimers. The proteins were tested against five bNAbs (VRC01, 2G12, PGT145, PGT128, PGT151).

To further assess the impact of antigenicity of our various protein productions, we used biolayer interferometry (BLI) to investigate their binding properties against the panel of bNAbs described in the above sections. For the BLI assays, the proteins purified with PGT145 immuno-affinity chromatography were used. We note that this purification method is selective for well-folded native-like trimers and deselects non-trimeric and otherwise misfolded protein species. High binding to quaternary- and glycan-dependent bNAb PGT151 suggested that some of the pre-fusion native-like features of the proteins produced from the three cell lines were preserved (Fig. 5). Our data clearly showed PGT145 binding was found to be variable, which could be attributed to differences in the conformation of the apex, but likely also differences in occupancy of apex PNGS. BG505 SOSIP.v4.1 trimers produced in the absence of either STT3A or STT3B did not display significant differences in binding to 2G12 and PGT128, irrespective of whether STT3A or STT3B was absent during production. In contrast, the binding of VRC01 was greater toward trimers produced in both STT3A-KO and STT3B-KO cells. In particular, this increase was highest for the STT3A-KO, which is consistent with the enhanced neutralization sensitivity observed (Fig. 3).

### Knocking out STT3A and STT3B leads to under-occupancy of specific PNGS

**Fig 6:**
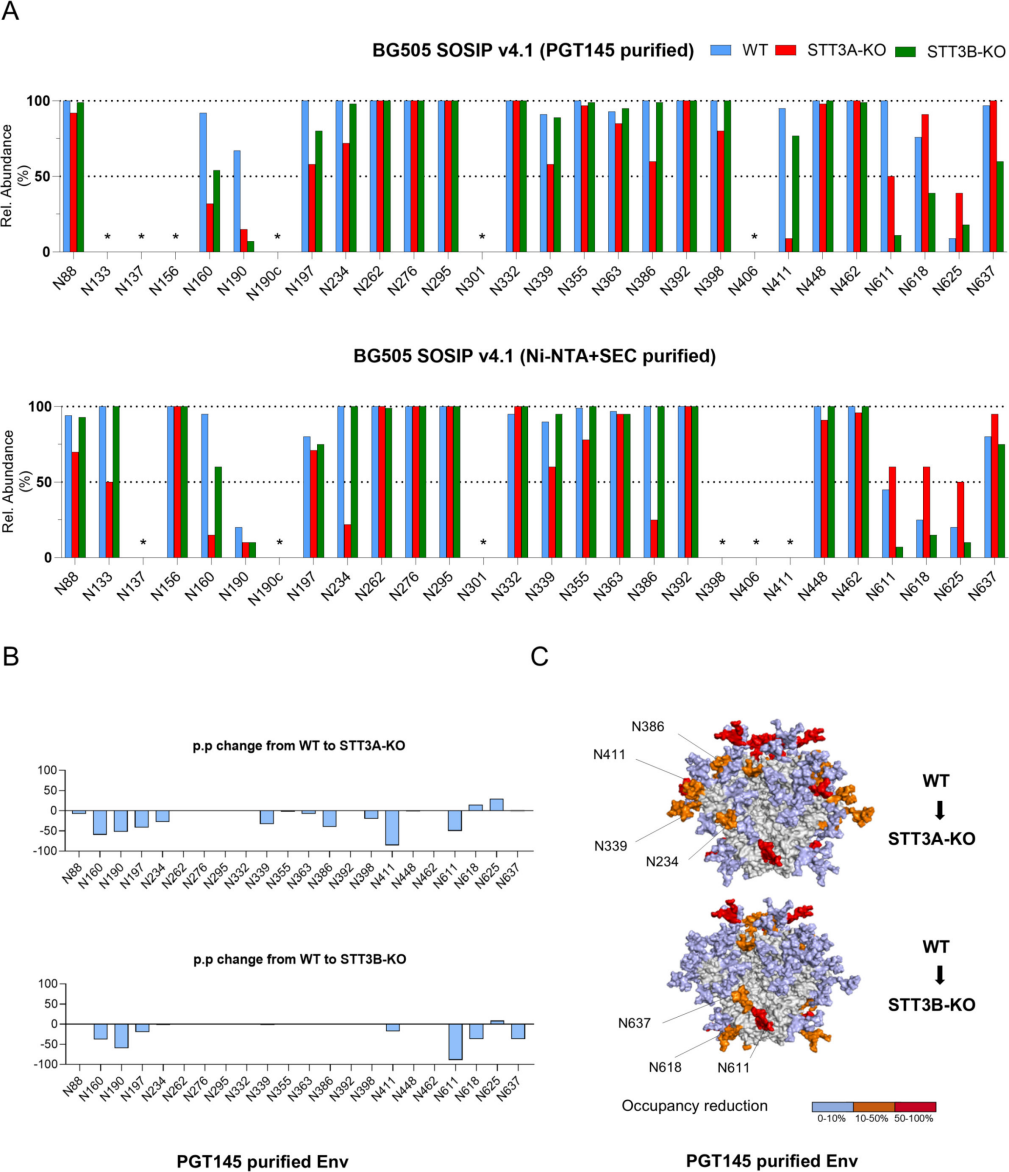
Glycan occupancy is decreased in STT3A-KO and STT3B-KO-produced BG505 soluble Env. (**A**) Quantification of site-specific occupancy for the 28 PNGS on Env trimers produced in different cell lines and analyzed by LC-ESI MS. The analysis included proteins purified via PGT145 immuno-affinity chromatography and those purified by Ni-NTA/SEC. Results are depicted as the mean of two independent biological replicates for each protein. The data displayed represents the PNGS occupancy expressed as the percentage of glycosylated peptide. ‘*’ indicates sites for which data could not be determined in at least one protein variant. (**B**) The presented data represent the arithmetic difference between the glycan occupancy of the STT3A- or STT3B-KO cell lines produced proteins minus the WT glycan occupancy, representing a percentage point change (p.p.). A negative p.p. change represents a lower occupancy of the KO variant compared to the WT. Only glycosylation sites for which data could be obtained for both WT and KO versions are included. (**C**) The structural models of the WT and KO Env trimers showing the glycan sites that were significantly impacted by STT3A and STT3B knockouts. Glycans are modeled onto each PNGS onto a previously generated BG505 structure (50). Colors indicate the degree of occupancy reduction from WT to STT3A/3B-KO: 0%–10% (light blue), 10%–50% (orange), and 50%–100% (red).

Liquid chromatography-electrospray ionization (LC-ESI) mass spectrometry (MS) with an Orbitrap Fusion mass spectrometer was used to determine PNGS occupancy on the Env trimers (8). PNGS occupancy is expressed as the percentage of the total peptide that is modified by a glycan. Comparing the WT PGT145- and Ni-NTA/SEC-purified proteins showed similar PNGS occupancy patterns with a few minor exceptions where PGT145-purification appeared to have selected for more occupied PNGS (e.g., N190, N611, and N618) (Fig. 6A). Eliminating STT3A or STT3B resulted in less PNGS occupancy across the Env trimer. However, most PNGS remained highly occupied. This observation could stem from redundancy between OST-A and OST-B, leading to one isoform compensating for the absence of the other. The impact of STT3A/B deletion is likely more significant than what is observed from material purified post-production, as highly unoccupied material is prone to aggregation and will likely be degraded inside the cell.

When studying PGT145-purified trimers, STT3A-KO led to reduced occupancy (>20% reduction compared to WT) at PNGS at N160, N190, N197, N234, N339, N386, N411, and N611, while STT3B-KO led to less occupancy at N160, N190, N197, N611, N618, and N637. A few general observations can be made. First, there is substantial overlap in the sites that are underoccupied in both cell lines, including sites that PNGS are closely spaced. For example, N160, which is in close proximity to N156, and N190, which is in close proximity to N190c, have reduced occupancy in both cell lines. N137, which is proximal to N133, also shows poor occupancy; however, the N137-containing glycopeptide was not resolved for the WT control. It is known that closely spaced PNGS are prone to under-occupancy (21), and our observations related to N160 and N190 suggest that this is exacerbated when STT3A or STT3B is absent. A decrease in occupancy at N197 was also observed in both KO cell lines (42% and 20% reductions for STT3A and STT3B-KO relative to WT, respectively), but the magnitude of this reduction was notably lower compared to that seen at N160 (60% and 38% reductions for STT3A and STT3B-KO relative to WT, respectively) and N190 (52% and 60% reductions for STT3A and STT3B-KO relative to WT, respectively) (Fig. 6A). A more detailed representation and the corresponding numerical quantification of occupancy at the 28 PNGS are provided in Fig. S2 and Table S1.

Subsequently, we studied whether particular glycosylation sites were dependent on either STT3A or STT3B. The glycan occupancy analysis showed that deletion of STT3A impacted glycosylation occupancy more globally than STT3B removal, even at sites, such as N234, N339, N386, and N411, which are usually fully occupied (8, 21). These sites, located on gp120 subunits, can be considered STT3A-dependent, as a similar reduction in occupancy was not observed in proteins produced in STT3B-KO cells. STT3B-KO largely impacted the occupancy of C-terminal glycan sites N611, N618, and N637, found in the gp41 ectodomain. This observation is in line with literature demonstrating that STT3B is mostly responsible for adding glycans near the C-termini of glycoproteins (12, 17). This phenomenon may be in part explained by C-terminal sequons appearing late during translation, therefore residing for a shorter time in the translocation channel and limiting exposure to STT3A. The site-specific occupancy data for the PGT145-purified trimers were subsequently plotted as percentage point changes from WT to KO cells to highlight the differences in glycan occupancy at specific sites for glycan sites where occupancy could be determined experimentally (Fig. 6B). To contextualize the changes in occupancy, we modeled glycans onto a previously generated WT structure (50) (Fig. 6C). Finally, site-specific N-glycan profiles of Env proteins indicated that STT3A/3B-KO did not result in a profound difference in glycan composition (Fig. S3).

## DISCUSSION

In this study, we investigated the role of STT3A and STT3B catalytic subunits on HIV-1 Env glycosylation in the context of Env-based vaccines. The OST-A and OST-B enzyme complexes, which contain STT3A and STT3B, respectively, add glycans to polypeptides cotranslationally or posttranslationally, respectively (14). It is now well known and studied that Env glycans hold a critical role in bNAb development during infection in people with HIV and that they have become a major focus of the HIV-1 vaccine design through their removal or addition to fill in glycan holes and/or create artificial glycan holes present on recombinant Env trimers used as immunogens to shape B cell responses toward neutralizing response (32, 38, 59, 60). However, the task is complex and controlling glycan occupancy often requires strain-specific engineering. Thus, developing new strategies may be needed to control glycan occupancy of Env-based vaccines. Defining the roles of N-linked glycosylation by STT3A and STT3B is beneficial in understanding the factors that result in the inefficient glycosylation of recombinant Env proteins. While the simultaneous knockout of STT3A and STT3B was not feasible because of cell death, the individual STT3A-KO or STT3B-KO cells helped to assess the role of each isoform in glycosylation. Overall, the current study demonstrated that the viral Env glycosylation is mostly controlled by and dependent on OST-A, and less so by OST-B. Although STT3A is primarily responsible for glycosylation of the viral Env, STT3B plays a comparatively greater role in the glycosylation of soluble Env immunogens. Furthermore, we found that the impact of STT3A/3B-KO on bNAb neutralization varied widely across different HIV-1 strains derived from different clades. Additionally, site-specific glycan analysis revealed that STT3A-KO results in a more global reduction in glycan occupancy, whereas STT3B plays a more specific role in the glycosylation of sites located closer to the C-terminus, consistent with studies on cellular glycoproteins (17).

Our data on viral production and infectivity support that the OST-A complex plays a more critical role in Env glycosylation than OST-B. In the context of full-length HIV-1 Env presented on virions, the transmembrane domain positions the gp41 glycans significantly upstream of the gp41 cytoplasmic tail C-terminus. As a result, these glycans are not located within the region most sensitive to the STT3B-specific glycosylation pathway, which preferentially acts on sites closer to the C-terminus (17). Thus, the spatial separation of gp41 glycan sites from the C-terminal region provides a plausible explanation for the minimal impact of STT3B KO on viral infectivity, as most of the gp41 glycosylation would be handled by STT3A. The strong dependence on STT3A and the lack of compensation from STT3B can also be explained by the membrane-bound nature of the viral Env glycoprotein and the lack of a free C-terminus in the lumen of the ER. Given that Env is a membrane-bound protein that folds cotranslationally, the rapid folding process and its association with the membrane may limit the ability of STT3B to access and glycosylate skipped sites. Additionally, the repositioning of acceptor sites away from the ER lumenal surface during folding may further hinder STT3B-mediated modification (18).

Viral production data indicated that STT3B-KO enhanced 398F1 and X1632-S2-B10 production dramatically. However, this effect was only partially reflected in infectivity, with 398F1 showing enhanced and X1632-S2-B10 showing no change in infectivity. Despite high levels of p24, infectivity remained low for the X1632-S2-B10 strain, suggesting that a substantial proportion of the produced virions were non-infectious. In the context of pseudoviruses, a high p24 signal may reflect non-functional particles, such as those with impaired Env incorporation or conformational defects (61, 62). As expected, p24-normalized infectivity data (Fig. S1A) confirmed reduced specific infectivity for both 398F1 and X1632-S2-B10 strains in STT3B-KO conditions. In contrast to these two strains, increased specific infectivity was observed for BG505, 246-F3_C10_2, and 25710-2-43 strains under STT3B-KO conditions despite no significant change in viral production (Fig. 2A and C; Fig. S1A). These strain-specific outcomes highlight the complex interplay between glycosylation, Env incorporation, and viral fitness. Another notable outlier is 398F1, in which infectivity was detected despite reduced p24 levels in STT3A-KO conditions. This observation shows that only a small fraction of infectious particles can be sufficient for a detectable infection signal in titration-based assays. Consistent with this, the p24-normalized infectivity data revealed that STT3A-KO caused reduced infectivity of 398F1 (Fig. S1A).

Another important consideration is that neutralization assays provide critical insights into the antigenic landscape of functional HIV-1 Env trimers. However, they provide information on functional Env only and offer no information about non-functional Env that may also be present on the viral surface. As a result, differences in glycosylation and/or epitope exposure that exist on non-functional Env remain undetected in standard neutralization readouts. Additional assays, such as binding to virion-derived Env or site-specific glycopeptide analysis, can provide complementary resolution (63–65).

We reported enhanced VRC01 binding to Env produced in the absence of STT3A and increased neutralization sensitivity of the pseudovirus generated in the absence of STT3A. Both results are consistent with improved accessibility of the CD4bs. We hypothesize that this is due to reduced occupancy of PNGS proximal to the CD4bs, in particular, the PNGS at N190, N197, N234, and N386. This is in line with observations that the absence of these sites improves epitope accessibility and increases neutralization sensitivity (66–70). Site-specific glycan analysis of the STT3A-KO Env protein supported this hypothesis, showing notable underoccupancy at positions N197 and N386 in particular.

In contrast, ablating STT3A activity conferred increased resistance to glycan-dependent bNAbs 2G12 and PGT128 for the BG505 strain. Here, we hypothesize that this resistance may result from underoccupancy at glycan sites that are part of the outer domain intrinsic mannose patch (48, 71, 72). Consistent with this observation, site-specific glycan analysis of the soluble BG505 SOSIP.v4.1 purified with PGT145 revealed reduced occupancy at several relevant PNGS, including N339 and N386, which are known to contribute to the epitopes recognized by V3-glycan-targeting bNAbs. Altogether, these findings suggest that STT3A-mediated glycosylation is critical for either shielding critical epitopes from antibody recognition or maintaining sensitivity to certain glycan-dependent antibodies, especially for BG505 Env protein and virus.

Site-specific glycan analysis of recombinant Env trimers showed that even when expressed in WT cells, purification strategy can significantly affect the glycan occupancy of soluble Env trimers. Notably, purification via PGT145 affinity chromatography led to >20% higher occupancy at glycan sites N190, N197, N611, and N618 compared to Ni-NTA/SEC purification when expressed in WT cells. As glycosylation is a critical determinant of Env antigenicity and immunogenicity, these differences underscore the importance of carefully selecting a purification method, particularly in the context of immunogen production.

Glycan analysis revealed that a large majority of the PNGS were still highly occupied from all producer cell lines tested. The ability to preserve glycan occupancy despite OST deficiencies highlights that the OST isoforms have redundant roles. This observation is, however, made for secreted purified glycoproteins, i.e., glycoproteins that have successfully completed the chaperone-assisted folding pathway. Our assays do not allow analysis of glycoproteins that did not fold properly because critical glycans were missing and were targeted for degradation (73, 74). Similarly, our viral production and infectivity analysis were performed on secreted viral particles. It is important to note that this may result in an overestimation of glycan occupancy as misfolded and/or under-glycosylated Env forms are less likely to be secreted or incorporated into virions. Another limitation of this study is that glycosylation profiles observed in HEK293T cells may not fully recapitulate those generated in primary infected cells, where differences in glycosylation machinery, expression levels, and cellular context can influence Env processing (8, 75). Additionally, given that STT3A and STT3B are key components of the N-glycosylation machinery, their knockout is likely to induce widespread changes in cellular protein processing and quality control (76). Such global modifications may indirectly influence viral protein synthesis, trafficking, and assembly, in addition to the direct effects on Env glycosylation examined here. While these indirect effects cannot be fully distinguished in the current experimental framework, they should be considered when interpreting the findings reported in this study.

Our results show that maximal glycosylation of Env involves the cooperation of both OST isoforms, and this is consistent with observations with other glycoproteins (14). Viral Env proteins are crucial for inducing neutralizing antibodies due to their critical involvement in viral entry and their potential as immunogen candidates (77–80). Therefore, a fundamental understanding of the role of OST complexes in viral envelope glycosylation could potentially lead to the development of new methods to mimic a fully glycosylated viral envelope and improve antigen quality, stability, and relevance. In HIV-1 vaccine research, the design of Env trimer immunogens has taken a central role with the aim of inducing broad and protective neutralizing antibody responses. To ensure that recombinant Env trimers mimic viral Env in terms of glycan occupancy, artificial glycan holes should be eliminated by increasing PNGS occupancy and membrane-bound immunogen approaches favored (8). These adjustments hold promise with regard to immunogen production batch-to-batch consistency, as well as controlling glycan holes, as needed to open and close areas on Env trimers to either enhance immune-focusing efforts or to further guide immune responses toward breadth mimicking antibody-virus co-evolution in people living with HIV that developed neutralization breadth and/or bNAbs linked to glycan shield evolutionary events. Revealing how OST-A and OST-B complexes contribute to the Env glycosylation offers valuable insights for immunogen design and will likely facilitate the development of methods, producer cells, and protein engineering to control glycan occupancy.

## MATERIALS AND METHODS

### Cell culture

Recombinant Env proteins and the infectious virus stocks were prepared by transfecting WT HEK293T (American Type Culture Collection Cat. #11268), as well as HEK293T STT3A-KO and STT3B-KO cell lines kindly provided by Dr. Reid Gilmore and Dr. Natalia Cherepanova. Cells were cultured under sterile conditions and kept at +37°C, 5% CO_2_, in a humidified incubator. Cell lines were maintained in culture using DMEM plus glutamate (GIBCO), and 10% fetal calf serum supplemented with antibiotics (i.e., penicillin and streptomycin, 100 U/mL each) (complete medium), and 0.05% wt/vol of Trypsin/EDTA solution was used to detach and passage the cells.

### BG505 intact virus and pseudovirus production

For the generation of intact virus stocks, WT HEK293T (2 × 10⁵), STT3A-KO (2.5 × 10⁵), and STT3B-KO (2 × 10⁵) cells were seeded in 3 mL/well of complete medium in 6-well tissue culture plates (Corning) to reach ~85%–90% confluency at the time of transfection. Cells were transfected with 5 µg of the previously described BG505 strain plasmid genomic DNA (21). To produce Env-pseudotyped viruses, WT and KO HEK293T cells were seeded at the same densities as for BG505 intact virus production. Transfections were carried out using 1.6 µg of a full-length Env expression plasmid and 2.4 µg of the Env-deficient HIV-1 backbone plasmid pSG5ΔEnv, as previously described (81). For both productions, plasmid DNA was diluted in 250 µL Opti-MEM (GIBCO) and mixed with 10 µL of Lipofectamine 2000 (Invitrogen) that had been pre-diluted in 240 µL Opti-MEM. After a 20-min incubation at room temperature, transfection mix solutions were added to the cells. Supernatants containing virus were harvested 48 h post-transfection and filtered through a 0.45 µm membrane for downstream use.

### p24 capsid (CA-p24) ELISA

To quantify HIV-1 particles, production of the capsid protein p24 (CA-p24) was assessed using the HIV-1 Gag p24 DuoSet ELISA kit (Bio-Techne, R&D Systems) following a custom in-house protocol (40). High binding half-area white 96-well plates (Greiner Bio-One) were coated with mouse anti-HIV-1 Gag CA-p24 capture antibody and incubated overnight at room temperature. The following day, plates were washed three times with wash buffer (1× PBS + 0.05% Tween 20) then blocked with 1% BSA in 1× PBS, 0.2% Triton X-100. Diluted samples, CA-p24 standard proteins, and controls (PBS) were added to the wells and incubated for 2 h at room temperature with continuous shaking. After incubation, the wells were washed again using washing buffer. A 1:80 dilution of Streptavidin-HRP (Bio-Techne, R&D Systems) was prepared using Reagent Diluent (Bio-Techne, R&D Systems) and added to the wells, followed by a 20-min incubation at room temperature. The plates were then washed again and tapped dry. Finally, a 1:10 dilution of LumiPhos A+B substrate was prepared using Milli-Q water and added to the wells for 2 min to allow signal development. Luminescence was measured immediately using a GloMax plate reader (Promega). Standard curves were generated, and data analysis was conducted to determine CA-p24 concentrations. The various productions of HIV-1 pseudovirus isolates and BG505 virus were measured in duplicate and in two independent ELISAs. p24 values shown were obtained from measurements within the assay’s linear range.

### Infectivity assay

TZM-bl cells (82) were seeded at a density of 1.7 × 10⁴ cells per well in 96-well plates 1 day before infection. Cells were cultured in complete medium and passaged as described above. On the following day, the harvested intact and pseudoviruses were titrated using TZM-bl cells and in quadruplicate as previously described (82). The TCID_50_ values were determined for each virus using GraphPad Prism v10.

### Antibody production

Recombinant antibodies were produced in HEK293F suspension cells as previously described (46). Briefly, heavy and light chain plasmid DNAs (156 μg each) were filtered and combined with polyethylenimine (PEI) MAX (Polysciences) at a 1:3 DNA:PEI ratio in Opti-MEM reduced serum medium (Gibco). The mixture was incubated for 30 min at room temperature before being added to HEK293F cultures. After a 6–7 day incubation, supernatants were collected, centrifuged, and filtered through 0.2 μm filters. Protein G coupled Agarose beads (Pierce, 20397) were added to the filtered supernatants, incubated overnight at +4°C with gentle rotation, the resin transferred to a centrifuge column (Pierce), washed twice with PBS (pH 7.2), and antibodies eluted using 0.1 M glycine (pH 2.5). The eluate was immediately neutralized using 1 M Tris (pH 8.6) as collection buffer in a 9:1 ratio. The eluate was concentrated and exchanged into 1× PBS using a 100 kDa molecular weight cut-off Vivaspins (Sartorius) and then passed through a 0.22 μm filter (Costar, 98231-UT-1). Final protein concentrations were determined by UV280 absorbance using the standard IgG extinction coefficient on a Nanodrop 2000 instrument (Thermo Scientific).

### Neutralization assay

TZM-bl cells were seeded at a density of 1.7 × 10⁴ cells per well in 96-well plates. The following day, the viral input equal to TCID_50_ for each virus was incubated for 60 min at room temperature using threefold serial dilutions of each bNAb tested. This mixture, supplemented with 40 µg/mL DEAE-dextran (40 μg/mL) and saquinavir (400 nM) at a 1:1 ratio, was added onto TZM-bl cells to a final volume of 200 µL/well. Three days post-infection, cells were washed with 1× PBS and lysed using lysis buffer (25 mM Glycylglycine [Gly-Gly], 15 mM MgSO_4_, 4 mM EGTA tetrasodium, 10% Triton X-100, pH 7.8). Bright-Glo kit (Promega, Madison, WI) was used to measure Luciferase activity. All infections were carried out in quadruplicate to ensure reproducibility. Background luminescence was subtracted using uninfected control wells. For normalization, the infectivity of each Env mutant in the absence of antibody was defined as 100%. Dose–response curves were generated using non-linear regression, and the half-maximal inhibitory concentration (IC₅₀) values were determined by fitting a sigmoidal curve in GraphPad Prism v10.

### Blue native-PAGE

To assess the correct folding into trimers of the produced proteins, these were subjected to a Blue native-polyacrylamide gel electrophoresis and visualized using colloidal blue stain (Life Technologies). Typically, 3 μg of purified protein was prepared in 4× MOPS buffer (200 mM MOPS, 200 mM Tris, pH 7.7) and resolved on a NuPAGE 4%–12% Bis-Tris gel (Novex). Electrophoresis was carried out for up to 60 min at 200 V using Invitrogen cathode (NB2001) and anode (NB2002) buffers. Gels were then stained with colloidal blue stain following the manufacturer’s instructions. Once destained with MiliQ water, the gels were imaged using a gel imaging system (Bio-Rad).

### Biolayer interferometry

Antibody binding to PGT145-purified Env trimers produced in WT, STT3A-KO, and STT3B-KO HEK293T cells was analyzed using a ForteBio Octet K2 instrument, as previously described (83). All measurements were performed at +30°C with shaking at 1,000 rpm. Antibodies and purified proteins were diluted in running buffer (1× PBS containing 0.1% BSA and 0.02% Tween-20) to a final volume of 300 μL/well. Protein A biosensors (ForteBio) were loaded with antibodies at a concentration of 2.0 μg/mL until a loading threshold of 0.5 nm was reached. Trimer proteins were prepared at 600 nM with association and dissociation phases monitored for 300 s each. Background binding was determined using sensors loaded with trimer plunged into running buffer in the absence of antibody.

### Env SOSIP trimers

The BG505 SOSIP.v4.1-derived trimers have been extensively described elsewhere, as have the methods to produce and purify them (57). Briefly, Env trimers were produced in WT HEK293T (15 × 10⁶), STT3A-KO (18.6 × 10⁶), and STT3B-KO (15 × 10⁶) cells seeded into multilayer T175 flasks (Thermo Scientific). Each flask was transfected with 180 μg of plasmid encoding the HIV-1 Env protein of interest and 50 μg of furin expression plasmid using PEI MAX. Following 72 h transient expression, supernatants were harvested and Env trimers purified either by PGT145-immunoaffinity chromatography (57) or Ni-NTA affinity chromatography, followed by SEC (Bio-Rad) to check for quality and isolate trimer peaks.

### Site-specific glycan analysis using mass spectrometry

Env protein samples (50 μg per aliquot) were denatured in 50 mM Tris-HCl (pH 8.0) containing 6 M urea and 5 mM dithiothreitol (DTT) for 1 h. Alkylation was performed by the addition of 20 mM iodoacetamide (IAA) for 1 h in the dark, followed by quenching with excess DTT. Samples were buffer-exchanged into 50 mM Tris-HCl (pH 8.0) using 10 kDa molecular weight cutoff centrifugal filters (Vivaspin) and subjected to overnight proteolytic digestion using trypsin, chymotrypsin, or α-lytic protease at an enzyme-to-substrate ratio of 1:30 (wt/wt), as previously described for site-specific glycoproteomic analyses (1–3).

Peptides were desalted using Oasis HLB 96-well solid-phase extraction plates and dried under vacuum prior to resuspension in 0.1% formic acid. Glycopeptides were analyzed by nano-liquid chromatography-electrospray ionization mass spectrometry using an Easy-nLC 1200 system coupled to an Orbitrap Fusion mass spectrometer. Peptide separation was performed on an EasySpray PepMap RSLC C18 analytical column (75 μm × 75 cm), with an in-line PepMap 100 C18 trap column (75 μm × 2 cm).

Peptides were resolved using a 275-min linear gradient from 0% to 32% acetonitrile in 0.1% formic acid over 240 min, followed by a high-organic wash. The flow rate was maintained at 200 nL min⁻¹. Data were acquired using stepped higher-energy collisional dissociation (HCD; 15%, 25%, and 45%), with MS1 scans collected at a resolution of 100,000 and MS2 scans at 30,000. Instrument parameters were set as previously reported for high-resolution glycopeptide analysis (21).

### Data processing of LC-MS data

Raw mass spectrometry files were processed using Byos software (Protein Metrics). Glycopeptide identification was performed using the Byonic search engine with precursor and fragment mass tolerances set to 4 and 10 ppm, respectively. Variable modifications included methionine oxidation, pyroglutamate formation from N-terminal glutamine or glutamate, deamidation of asparagine, and carbamidomethylation of cysteine.

Protease-specific searches were conducted separately for each digest using appropriate cleavage rules (trypsin RK, chymotrypsin YFW, α-lytic protease TASV), allowing for semi-specific cleavage and up to two missed cleavages. Peptide-spectrum matches were filtered to a 1% false discovery rate. Following identification, data sets from all protease digests were merged for downstream quantitative analysis.

Quantification was performed by summing extracted ion chromatogram areas across all observed charge states for each glycopeptide. Site-specific glycan distributions and site occupancy were calculated by comparing the relative abundances of different glycoforms sharing the same peptide backbone. All glycopeptide assignments were manually validated based on the presence of peptide backbone fragment ions (b/y ions) and diagnostic glycan oxonium ions, consistent with established glycoproteomic workflows (21, 84).

## Data Availability

All data needed to evaluate the results are included in the article and its supplemental material.

## SUPPLEMENTAL MATERIAL

Supplemental material jvi.01481-25-s0001.docx Fig. S1 to S3; Table S1.

ASM does not own the copyrights to Supplemental Material that may be linked to, or accessed through, an article. The authors have granted ASM a non-exclusive, world-wide license to publish the Supplemental Material files. Please contact the corresponding author directly for reuse.
