# Supplementary material for: Differential contributions of human oligosaccharyltransferase complexes OST-A and OST-B to HIV-1 envelope glycoprotein glycosylation
Source: J Virol. 2026 Mar 4;100(3):e01481-25. doi: 10.1128/jvi.01481-25 (PMC13011424; doi:10.1128/jvi.01481-25)
Supplement: Supplemental material — Fig. S1 to S3; Table S1. [file jvi.01481-25-s0001.docx]

**Supplementary Materials**

**
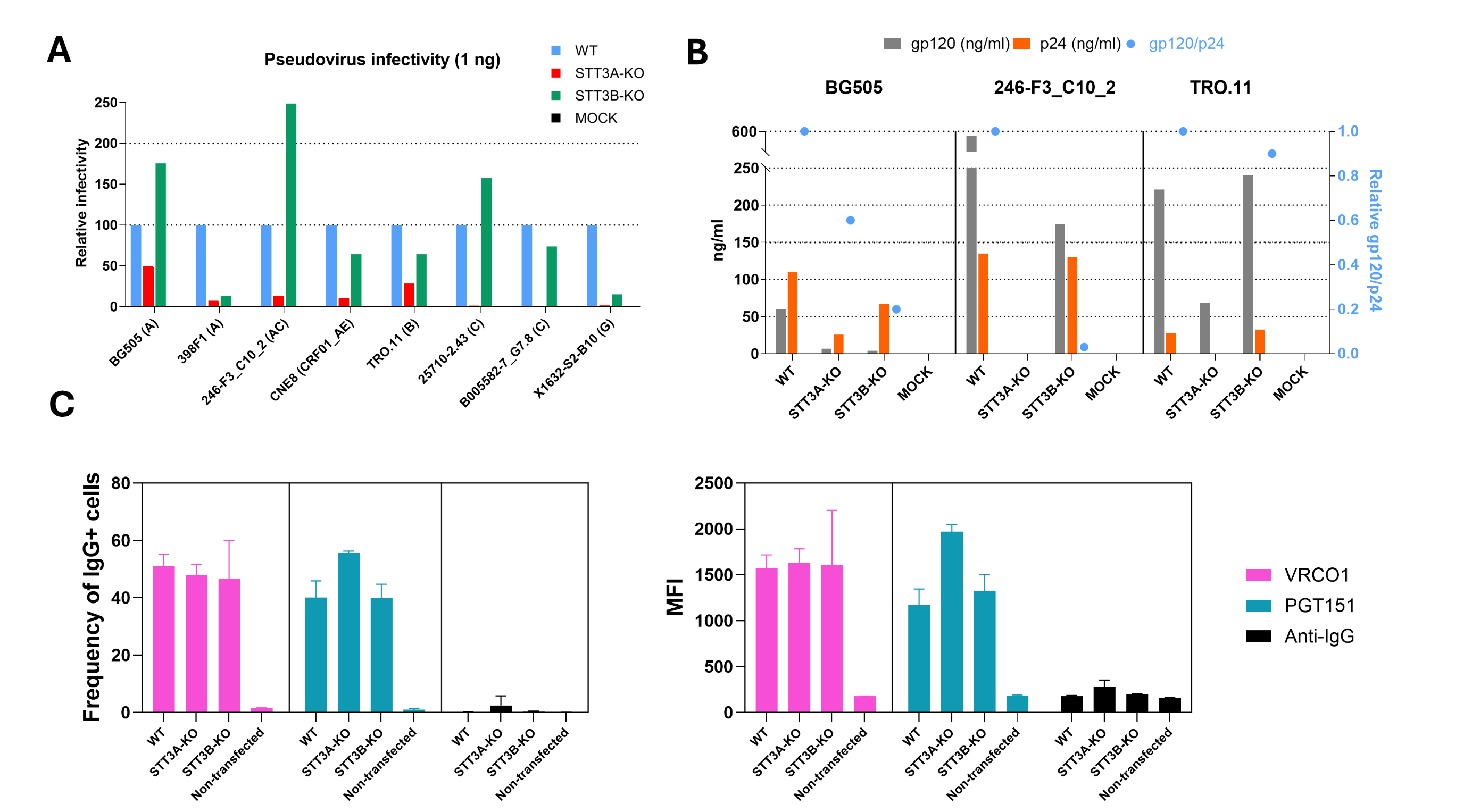
**

**Figure S1. (A) Relative infectivity of pseudoytyped HIV-1 viruses produced in different cell types.** Infections were performed using 1 ng of each pseudovirus. The measured luciferase activity was normalized to that of the corresponding WT virus. **(B) Quantification of gp120 and p24 levels by ELISA from the supernatants of pseudovirus-producing cells.** The right Y axis shows the relative gp120/p24 ratio, normalized to the corresponding WT condition, which was set to 1. **(C)** **Cell surface Env expression, assessed by flow cytometry using HIV-1 broadly neutralizing antibodies.** Data are shown as frequency of Env-positive cells and mean fluorescence intensity (MFI), as indicated.


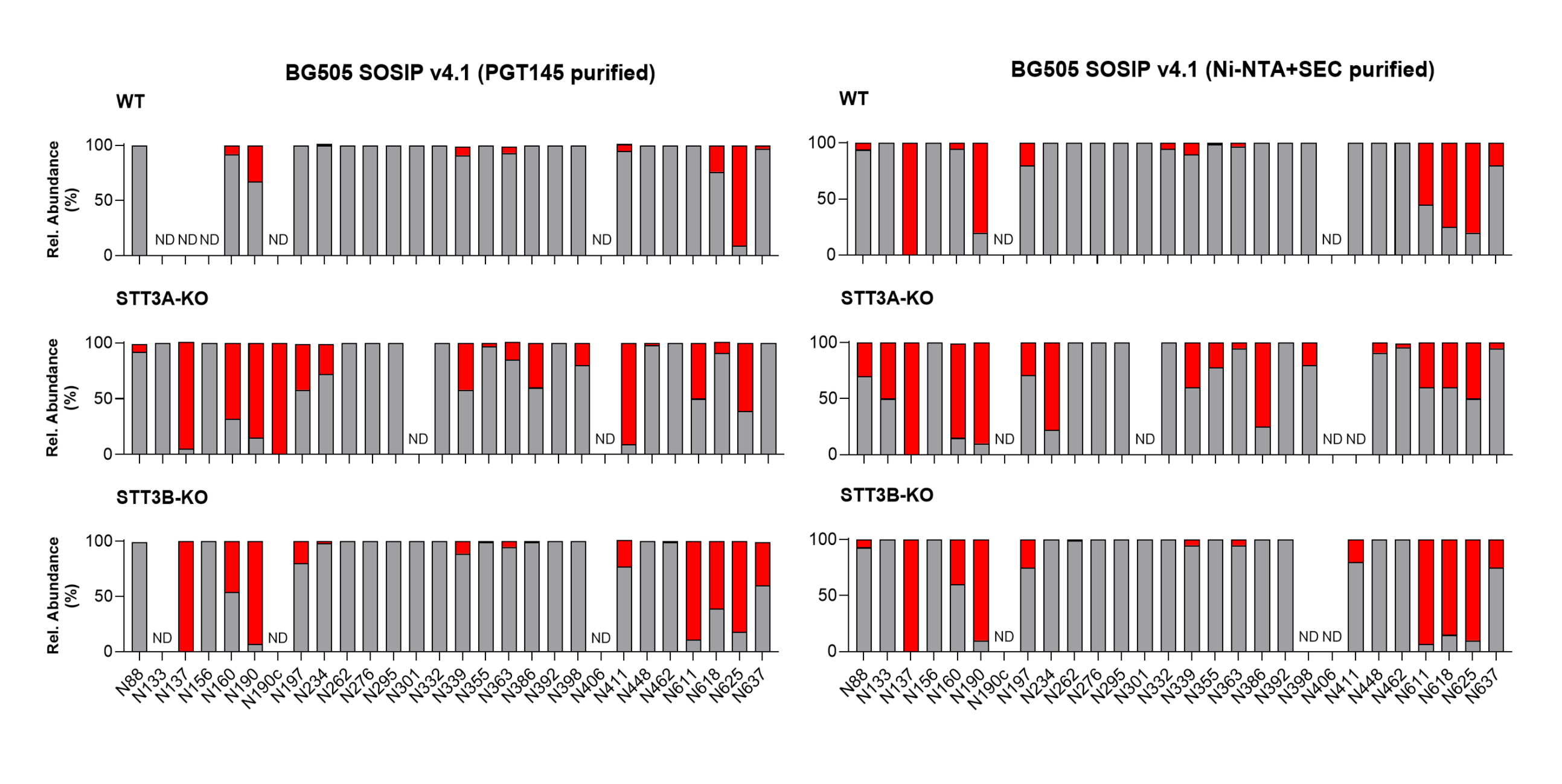


**Figure S2. Site-specific glycan occupancy of BG505 Env trimers. Related to Figure 6A.** BG505 SOSIP v4.1 proteins were purified either by PGT145 immuno-affinity chromatography or by Ni-NTA followed by SEC. Quantification of site-specific glycan occupancy at the 28 PNGS of Env trimers expressed in different cell lines was analyzed by LC-ESI MS. Values represent the mean from two independent biological replicates for each protein. The data displayed represents the relative proportion of occupied (grey) versus unoccupied (red). ‘ND’ indicates the sites where data could not be obtained. A summarized representation of these data is shown in Figure 6.


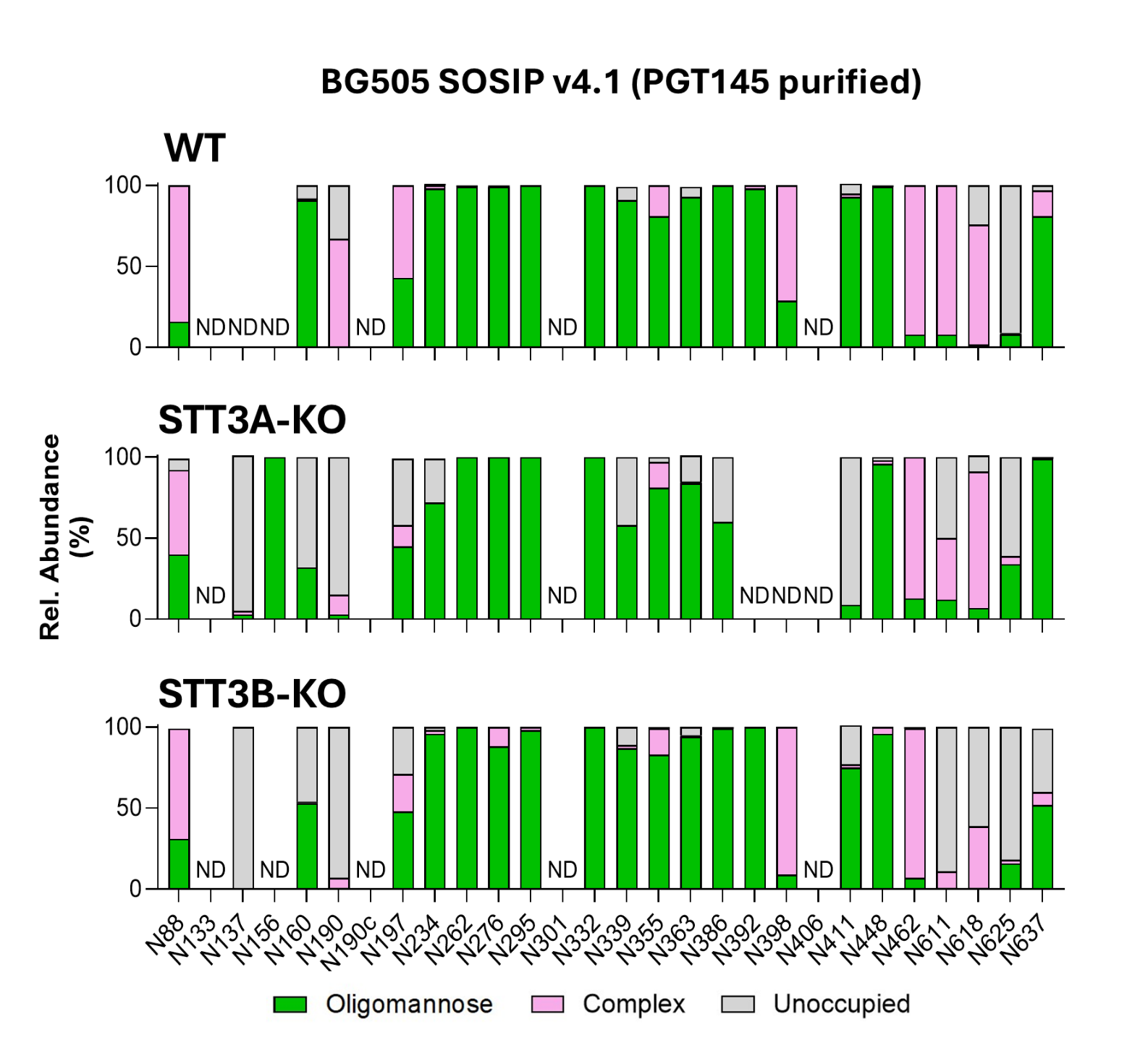


**Figure S3.** **Site-specific glycan analysis of BG505 proteins produced in WT, STT3A-KO, and STT3B-KO HEK293T cells.** BG505 SOSIP v4.1 proteins were purified by PGT145 immuno-affinity chromatography NTA followed by SEC. Values represent the mean from two independent biological replicates for each protein. The oligomannose-type glycans are shown in green and complex glycans are shown in pink. Unoccupancy of an N-linked glycan site is represented in gray. ‘ND’ indicates the sites for which data could not be determined.

**Table S1. Site-specific glycan occupancy of BG505 Env trimers. Related to Figure 6 A&B.** Detailed numerical values corresponding to the quantification of occupancy at the 28 PNGS (shown in Supplementary Figure 3) are presented, together with the calculated percentage point (p.p.) changes (Figure 6B). Light grey shading indicates sites where occupancy data could not be detected. Dark grey shading denotes cases where percentage point changes could not be calculated due to missing occupancy data.

|  | BG505 SOSIP v4.1 (PGT145 purified) | | | | | BG505 SOSIP v4.1 (Ni-NTA+SEC purified) | | | | |
| --- | --- | --- | --- | --- | --- | --- | --- | --- | --- | --- |
|  | Glycan occupancy | | | p.p. change | | Glycan occupancy | | | p.p. change | |
|  | WT | STT3A-KO | STT3B-KO | WT to STT3A-KO | WT to STT3B-KO | WT | STT3A-KO | STT3B-KO | WT to STT3A-KO | WT to STT3B-KO |
| N88 | 100 | 92 | 99 | -8 | -1 | 94 | 70 | 93 | -24 | -1 |
| N133 |  | 100 |  |  |  | 100 | 50 | 100 | -50 | 0 |
| N137 |  | 5 | 0 |  |  | 0 | 0 | 0 | 0 | 0 |
| N156 |  | 100 | 100 |  |  | 100 | 100 | 100 | 0 | 0 |
| N160 | 92 | 32 | 54 | -60 | -38 | 95 | 15 | 60 | -80 | -35 |
| N190 | 67 | 15 | 7 | -52 | -60 | 20 | 10 | 10 | -10 | -10 |
| N190c |  | 0 |  |  |  |  |  |  |  |  |
| N197 | 100 | 58 | 80 | -42 | -20 | 80 | 71 | 75 | -9 | -5 |
| N234 | 100 | 72 | 98 | -28 | -2 | 100 | 22 | 100 | -78 | 0 |
| N262 | 100 | 100 | 100 | 0 | 0 | 100 | 100 | 99 | 0 | -1 |
| N276 | 100 | 100 | 100 | 0 | 0 | 100 | 100 | 100 | 0 | 0 |
| N295 | 100 | 100 | 100 | 0 | 0 | 100 | 100 | 100 | 0 | 0 |
| N301 | 100 |  | 100 |  | 0 | 100 |  | 100 |  | 0 |
| N332 | 100 | 100 | 100 | 0 | 0 | 95 | 100 | 100 | 5 | 5 |
| N339 | 91 | 58 | 89 | -33 | -2 | 90 | 60 | 95 | -30 | 5 |
| N355 | 100 | 97 | 99 | -3 | -1 | 99 | 78 | 100 | -21 | 1 |
| N363 | 93 | 85 | 95 | -8 | 2 | 97 | 95 | 95 | -2 | -2 |
| N386 | 100 | 60 | 99 | -40 | -1 | 100 | 25 | 100 | -75 | 0 |
| N392 | 100 | 100 | 100 | 0 | 0 | 100 | 100 | 100 | 0 | 0 |
| N398 | 100 | 80 | 100 | -20 | 0 | 100 | 80 |  | -20 |  |
| N406 |  |  |  |  |  |  |  |  |  |  |
| N411 | 95 | 9 | 77 | -86 | -18 | 100 |  | 80 |  | -20 |
| N448 | 100 | 98 | 100 | -2 | 0 | 100 | 91 | 100 | -9 | 0 |
| N462 | 100 | 100 | 99 | 0 | -1 | 100 | 96 | 100 | -4 | 0 |
| N611 | 100 | 50 | 11 | -50 | -89 | 45 | 60 | 7 | 15 | -38 |
| N618 | 76 | 91 | 39 | 15 | -37 | 25 | 60 | 15 | 35 | -10 |
| N625 | 9 | 39 | 18 | 30 | 9 | 20 | 50 | 10 | 30 | -10 |
| N637 | 97 | 100 | 60 | 3 | -37 | 80 | 95 | 75 | 15 | -5 |
|  |  |  |  |  |  |  |  |  |  | Not detected |
|  |  |  |  |  |  |  |  |  |  | Not applicable |
